# High-throughput nanopore targeted sequencing for efficient drug resistance assay of *Mycobacterium tuberculosis*

**DOI:** 10.3389/fmicb.2024.1331656

**Published:** 2024-05-22

**Authors:** Chen Tang, Lianpeng Wu, Machao Li, Jianyi Dai, Ye Shi, Qiongdan Wang, Feng Xu, Laibao Zheng, Xingxing Xiao, Junwen Cai, Yanjun Zhang, Yuting Yang, Xiaoqun Zheng, Guangxin Xiang

**Affiliations:** ^1^School of Laboratory Medicine and Life Sciences, Wenzhou Medical University, Wenzhou, Zhejiang, China; ^2^Key Laboratory of Laboratory Medicine, School of Laboratory Medicine and Life Sciences, Wenzhou Medical University, Wenzhou, Zhejiang, China; ^3^Department of Clinical Laboratory, Wenzhou Central Hospital, Wenzhou, Zhejiang, China; ^4^National Key Laboratory of Intelligent Tracking and Forecasting for Infectious Diseases, National Institute for Communicable Disease Control and Prevention, Chinese Center for Disease Control and Prevention, Beijing, China; ^5^Department of Infectious Diseases, Wenzhou Central Hospital, Wenzhou, Zhejiang, China; ^6^Department of Clinical Laboratory, The First Affiliated Hospital of Wenzhou Medical University, Wenzhou, Zhejiang, China

**Keywords:** *Mycobacterium tuberculosis*, drug resistance, drug susceptibility testing, nanopore sequencing, targeted sequencing, multiplex PCR

## Abstract

Drug-resistant tuberculosis (TB), especially multidrug-resistant tuberculosis (MDR-TB) and extensively drug-resistant tuberculosis (XDR-TB), is one of the urgent clinical problems and public health challenges. Culture-based phenotypic drug susceptibility testing (pDST) is time-consuming, and PCR-based assays are limited to hotspot mutations. In this study, we developed and validated a convenient and efficient approach based on high-throughput nanopore sequencing technology combined with multiplex PCR, namely nanopore targeted sequencing (NTS), to simultaneously sequence 18 genes associated with antibiotic resistance in *Mycobacterium tuberculosis* (MTB). The analytical performance of NTS was evaluated, and 99 clinical samples were collected to assess its clinical performance. The NTS results showed that MTB and its drug resistance were successfully identified in approximately 7.5 h. Furthermore, compared to the pDST and Xpert MTB/RIF assays, NTS provided much more drug resistance information, covering 14 anti-TB drugs, and it identified 20 clinical cases of drug-resistant MTB. The mutations underlying these drug-resistant cases were all verified using Sanger sequencing. Our approach for this TB drug resistance assay offers several advantages, including being culture-free, efficient, high-throughput, and highly accurate, which would be very helpful for clinical patient management and TB infection control.

## Introduction

Before the outbreak of COVID-19, tuberculosis (TB) was reported to be the leading cause of human death due to infection, with 1.7 million deaths per year worldwide, (Daley, 2019). The causative agents of TB are known as the *Mycobacterium tuberculosis* complex (MTBC), which is a group of closely related bacteria and has a very complicated drug resistance spectrum (Gagneux, 2018). Humans are susceptible to eight MTBC members, namely *Mycobacterium tuberculosis* (MTB), *Mycobacterium bovis*, *Mycobacterium bovis* BCG, *Mycobacterium africanum*, *Mycobacterium canettii*, *Mycobacterium microti*, *Mycobacterium caprae*, and *Mycobacterium orygis*, the first of which is the main causative agent for TB, resulting in high morbidity and mortality globally (Esteban and Muñoz-Egea, 2016; Riojas et al., 2018; Kanipe and Palmer, 2020; Kanabalan et al., 2021).

MTB acquires antibiotic resistance through gene mutation, which has a great impact on clinical treatment (Koch et al., 2018; Singh and Chibale, 2021; Salari et al., 2023). Affected by COVID-19, a large number of new TB patients were not diagnosed and treated in time (Huang and Zhao, 2022). These MTB infections, especially multidrug-resistant (MDR) and extensively drug-resistant (XDR) strains, have created an urgent need for improved detection tools to guide TB treatment options.

TB surveillance of highly virulent and MDR strains is paramount for adequate diagnosis and treatment (CRyPTIC Consortium and the 100,000 Genomes Project et al., 2018). Currently, culture-based phenotypic drug susceptibility testing (pDST) is commonly used for assaying MTB, which usually takes weeks (Koch et al., 2018). Recommended by the World Health Organization, the real-time PCR-based Xpert MTB/RIF assay is much more efficient but limited to hotspot mutations, providing only partial data on drug resistance (Weinrick, 2020). With the advantages of high throughput and sensitivity, the targeted next-generation sequencing (tNGS) technology analyzes DNA information to determine the typing of drug resistance without culturing MTB (Murphy et al., 2023; Wu et al., 2023), and the nanopore sequencing newly introduced has the potential to quickly assay MTB (Cabibbe et al., 2020; Cervantes et al., 2020; Hu et al., 2021; Dippenaar et al., 2022).

To simultaneously assay the drug resistance of MTB against first-line and second-line anti-TB drugs, in this study, we developed a method based on nanopore sequencing technology combined with multiplex PCR, namely nanopore targeted sequencing (NTS). After the assessment of analytical performance and turnaround time (TAT), we further evaluated NTS to determine sensitivity, specificity, positive and negative predictive values, and consistency with the pDST and Xpert MTB/RIF assays through clinical samples.

## Materials and methods

### Samples

The reference strain MTB H37Rv (GenBank accession no. NC_000962.3) was purchased from Bangdesheng Biotechnology (Guangdong, China). Four MTB isolates, three strains of MTBC members, namely *Mycobacterium bovis*, *Mycobacterium bovis* BCG, and *Mycobacterium africanum*, and five strains of non-tuberculous mycobacteria (NTM) species, namely *Mycobacterium avium*, *Mycobacterium intracellulare*, *Mycobacterium abscessus*, *Mycobacterium kansasii*, and *Mycobacterium chelonae*, were provided by the National Institute for Communicable Disease Control and Prevention, Chinese Center for Disease Control and Prevention (China CDC, Beijing, China). Three other pathogens, namely *Streptococcus agalactiae*, *Streptococcus pneumoniae*, and *Escherichia coli*, were purchased from ATCC (Manassas, VA).

A total of 99 clinical samples were collected from Wenzhou Central Hospital. The study design was approved by the Research Ethics Board of Wenzhou Central Hospital, and all analyses were performed following the Declaration of Helsinki (L2023-02-033).

### DNA extraction

Genomic DNA was extracted using the boiling method for isolates and clinical samples. Briefly, 1 mL of sample liquid was centrifuged at 12,000 rpm for 10 min to remove the supernatant and resuspended in 50 μL of PBS, followed by adding 50 μL of nucleic acid extraction reagent (10 mM Tris–HCl, 1 mM EDTA, 1% TritonX-100, 1% NP-40, and 50% Chelex-100). The mixture was then boiled at 100°C for 10 min and centrifuged at 12000 rpm for 5 min, and finally, 10 μL of supernatant was taken as the template for multiplex PCR. For clinical samples such as sputum and bronchoalveolar lavage fluid (BALF), they were pre-treated with 4% NaOH and PBS solutions.

### Multiplex PCR and library preparation

As genome sequences of MTBC members are almost identical with more than 99% similarity (Kanabalan et al., 2021), primers were designed for the 8 MTBC genomes with their 18 drug resistance-associated genes, including *gyrB*, *gyrA*, *rpoB*, *mmpR5*, *rpsL*, *rplC*, *atpE*, *rrs* (16S rRNA gene), *rrl*, *fabG1*, *inhA*, *rpsA*, *tlyA*, *katG*, *pncA*, *eis*, *embB*, and *ubiA*. Several sets of primers were initially screened and checked by the quality control tool MFEprimer (iGeneTech Bioscience, Beijing, China) ^1^with the parameters of Tm 59°C and dNTPs 0.2 mM (Wang et al., 2019), and synthesized by Shenggong Biotechnology (Shanghai, China). The optimal primer set was experimentally validated, as were the primer concentrations. A single-tube multiplex PCR was developed for simultaneous amplification of these 18 genes.

A total volume of 25 μL PCR reaction was set up that included Q5 Hot-start high-fidelity DNA polymerase (New England Biolabs, Ipswich, MA), High GC enhancer, reaction buffer, dNTPs, multi-primer mixture, and the template, followed by a standard 3-step PCR protocol: initial denaturation at 98°C for 2 min; 40 cycles at 95°C for 25 s, 60°C for 30 s, and 72°C for 3 min; and a final step of 72°C for 4 min. After a 1% agarose gel electrophoresis for 2 h at a constant 120 V, the amplicons were visualized under UV transillumination.

The concentration of amplicons was determined using a Qubit dsDNA HS kit and measured with a Qubit 4.0 fluorimeter (Thermo Fisher Scientific, Waltham, MA), followed by purification using 1.2× AMPure XP beads (Beckman Coulter, Brea, CA). According to the manufacturer’s protocols, the DNA was end-repaired and dA-tailed using the NEBNext Ultra II end repair/dA-tailing module (New England Biolabs), followed by ligation with barcodes using the Blunt/TA ligation master mix (New England BioLabs) and Native Barcoding Kit (Oxford Nanopore Technologies (ONT), Oxford, UK). The pooled library was adapter-ligated using a Sequencing Ligation Kit (ONT). Prior to each step of the reactions, DNA was purified using 1.2× AMPure XP beads (Beckman Coulter), washed twice with 80% ethanol, and eluted with nuclease-free water.

### Nanopore sequencing

The purified library was loaded onto the flow cell R9.4.1 (FLO-PRO002, ONT) or flow cell R10.4.1 (FLO-PRO114M, ONT). Sequencing on PromethION (ONT) took 1 or 3 h upon request, with the operating software MinKNOW (ONT) driving the device. The Sequencing run was performed by Benagen Technology (Hubei, China).

### Bioinformatics analysis

Real-time data acquisition was performed with MinKNOW and stored in FAST5 file format, which was then converted into raw sequence data using the Albacore base recognition analysis and stored in FASTQ file format. There is a certain error rate during the sequencing proce*s*s, and the Phred quality value can measure the probability of incorrect detection of bases. Reads with a Phred quality value of ≤7 (R9.4.1) or ≤ 9 (R10.4.1) were filtered out using NanoFilt. The detection of mutations conferring antibiotic drug resistance was conducted through the bioinformatic pipeline TBProfiler, with mutation libraries updated regularly (Mahe et al., 2019; Phelan et al., 2019). Available on GitHub,^2^ TBProfiler was installed through the Bioconda channel for the interpretation of the drug resistance profile.

### Limit of detection (LOD)

The MTB H37Rv was diluted from 10^7^ bacteria/mL in a series of 10-fold gradients to 10^2^ bacteria/mL. The sequencing depths corresponding to different MTB concentrations were analyzed at five sequencing time points, including 15 min, 30 min, 60 min, 120 min, and 180 min. By mapping output reads to the TB reference genome of MTB H37Rv, reads with >90% identity were calculated. As time accumulated, the mapped number of reads in each sample increased, as did the sequencing depths for each gene. The LOD was defined as the concentration at which the sequencing depths of each gene were greater than 100 ×.

### pDST assay

According to the Clinical and Laboratory Standards Institute’s recommendations, pDSTs were conducted using two methods. For first-line agents including rifampicin (RIF) (1 μg/mL), isoniazid (INH) (0.1 μg/mL), ethambutol (EMB) (5 μg/mL), and streptomycin (STR) (1 μg/mL), the pDSTs were performed using liquid (MGIT 960 system and BACTEC MGIT 960 SIRE kit) (Becton Dickinson Diagnostic System, Sparks, MD) (Siddiqi et al., 2012). For second-line agents including amikacin (1 μg/mL) and fluoroquinolones (FQ) (1 μg/mL of levofloxacin and 1 μg/mL of moxifloxacin), the absolute concentration method was used to determine drug susceptibility patterns (Koh et al., 2012).

### Xpert MTB/RIF assay

According to the manufacturer’s instructions, Xpert Sample Reagent (Cepheid, Sunnyvale, CA) was added to the unprocessed sputum sample in a 2:1 ratio into a 15-ml centrifuge tube and incubated at room temperature for 15 min. During incubation, the samples were mixed by inverting the tubes two times every 5 min. Then, 2 mL of liquefied sample was transferred to Xpert Cartridge and loaded into GeneXpert IV machine (Cepheid). Within 2 h, the Xpert machine provided the results.

### Sanger sequencing

Sanger sequencing was performed using the ABI PRISM 3730 DNA Sequencer (Applied Biosystems, Foster, CA), which was run by Shenggong Biotechnology.

## Results

### NTS process establishment

**Figure 1:**
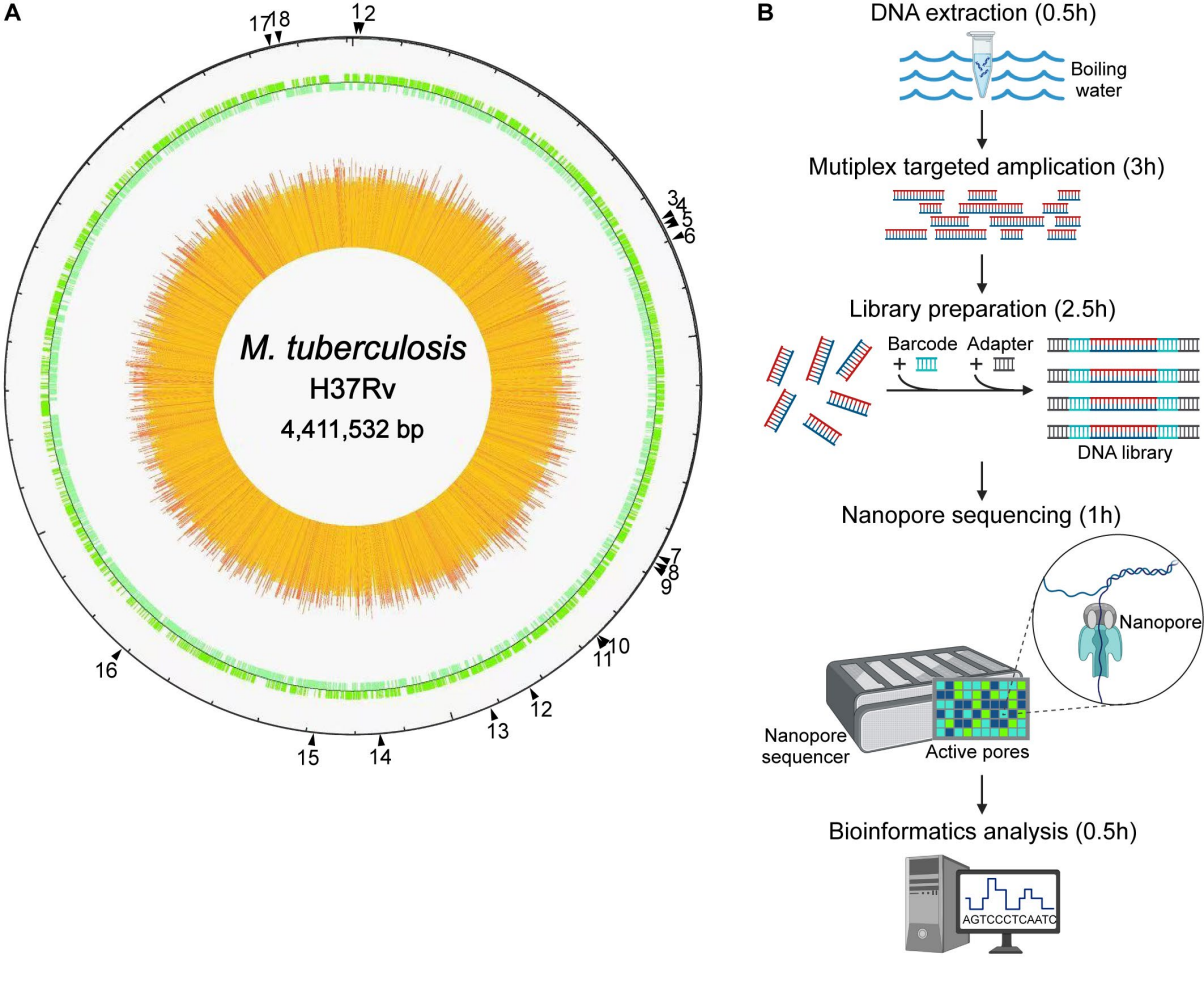
Nanopore targeted sequencing process for drug resistance assay of *Mycobacterium tuberculosis* (MTB). **(A)** The drug resistance-associated genes marked by black triangles with their positions in the genome of the reference strain MTB H37Rv, including 18 highly recommended genes which were *gyrB*, *gyrA*, *rpoB*, *mmpR5*, *rpsL*, *rplC*, *atpE*, *rrs*, *rrl*, *fabG1*, *inhA*, *rpsA*, *tlyA*, *katG*, *pncA*, *eis*, *embB*, and *ubiA*, respectively. **(B)** Process of high-throughput nanopore sequencing combined with single-tube multiplex PCR-based target enrichment. After DNA extraction, the targeted regions of drug resistance-associated genes were amplified by multiplex PCR, followed by barcoding and adapter ligation, and the prepared library was then loaded onto the flow cell on the nanopore sequencer, which generated long-read sequence data finally analyzed with bioinformatics. The turnaround time of the NTS process was approximately 7.5 h.

As shown in Figure 1, the NTS process for drug resistance assay of MTB was established, with 18 genes associated with MTB drug resistance, namely *gyrB*, *gyrA*, *rpoB*, *mmpR5*, *rpsL*, *rplC*, *atpE*, *rrs*, *rrl*, *fabG1*, *inhA*, *rpsA*, *tlyA*, *katG*, *pncA*, *eis*, *embB,* and *ubiA*, according to previous reports in this field (Zhao et al., 2014; Tafess et al., 2020; Liang et al., 2021; Wang et al., 2021; Dippenaar et al., 2022; Liu et al., 2022; Shi et al., 2022). High-throughput long-read nanopore sequencing was combined with the boiling DNA extraction method, the single-tube multiplex PCR, and the bioinformatic pipeline TBProfiler, and the turnaround time of this NTS was only approximately 7.5 h (Figure 1B).

### NTS analytical performance

**Figure 2:**
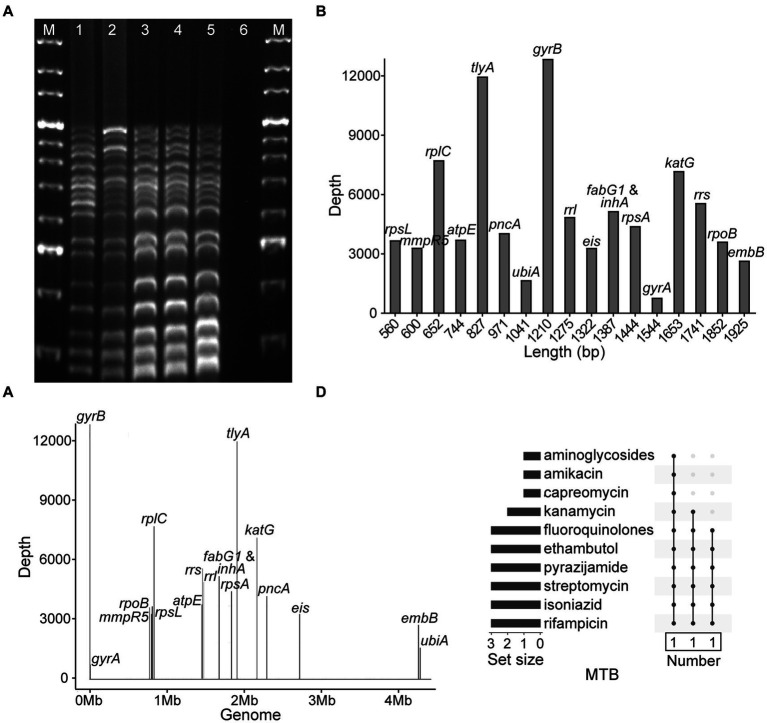
Development of nanopore targeted sequencing with multiplex PCR for drug resistance assay of *Mycobacterium tuberculosis* (MTB). **(A)** Electrophoresis of multiplex PCR-based amplicons from five MTB samples and a negative control. The bands of electrophoretic lanes 1–5 from bottom to top were *rpsL* (560 bp), *mmpR5* (600 bp), *rplC* (652 bp), *atpE* (744 bp), *tlyA* (827 bp), *pncA* (971 bp), *ubiA* (1,041 bp), *gyrB* (1,210 bp), *rrl* (1,275 bp), *eis* (1,322 bp), *fabG1* and *inhA* (1,387 bp), *rpsA* (1,444 bp), *gyrA* (1,544 bp), *katG* (1,653 bp), *rrs* (1741 bp), *rpoB* (1845 bp), and *embB* (1925 bp), respectively. The ladder sizes of DNA markers (M) were from 600 bp to 4,000 bp. The negative control was nuclease-free water. **(B)** Bar chart of 18 drug resistance-associated genes arranged according to the sizes of gene fragments against the sequencing depth of each gene after nanopore sequencing. **(C)** Distribution of 18 drug resistance-associated genes in MTB genome with their corresponding sequencing depths. **(D)** NTS-based drug resistance data are available for isolates after using the TBProfiler. Each row is a drug, and the columns with filled cells represent the set of isolates possessing drug resistance information. The box below shows the number of isolates in the set. The bar plot in the left panel shows the number of isolates with resistance information for each drug.

To evaluate the accuracy and specificity of NTS, the MTB H37Rv, four MTB isolates, three isolates of MTBC members, five strains of NTM species, and three other pathogens were assayed, and the results demonstrated that NTS successfully discriminated MTBC from NTM, other bacteria (Table 1; Figure 2A; Supplementary Figure S1; Supplementary Table S1), and multiple bacteria (data not shown). Multiplex PCR-based amplicons could be visualized using agarose gel electrophoresis, showing that MTBC possessed 17 bands from bottom to top: *rpsL* (560 bp), *mmpR5* (600 bp), *rplC* (652 bp), *atpE* (744 bp), *tlyA* (827 bp), *pncA* (971 bp), *ubiA* (1,041 bp), *gyrB* (1,210 bp), *rrl* (1,275 bp), *eis* (1,322 bp), *fabG1* and *inhA* (1,387 bp), *rpsA* (1,444 bp), *gyrA* (1,544 bp), *katG* (1,653 bp), *rrs* (1741 bp), *rpoB* (1845 bp), and *embB* (1925 bp), respectively. As *fabG1* is very close to *inhA* in the MTB genome, they were amplified with the same primer pair.

**Table 1:** Reference strain MTB H37Rv and four isolates of *Mycobacterium tuberculosis* (MTB), three isolates from the MTB complex, and five strains of non-tuberculous mycobacteria for the establishment of nanopore targeted sequencing.

No.	Strain name	Strain type	Drug resistance
1	MTB	Reference strain	Susceptible
2	MTB	Isolate	Susceptible
3	MTB	Isolate	XDR^#^
4	MTB	Isolate	XDR^$^
5	MTB	Isolate	XDR^&^
6	*M. bovis*	Isolate	Susceptible
7	*M. bovis* BCG	Isolate	Susceptible
8	*M. africanum*	Isolate	Susceptible
9	*M. avium*	Isolate	/
10	*M. intracellulare*	Isolate	/
11	*M. abscessus*	Isolate	/
12	*M. kansasii*	Isolate	/
13	*M. chelonae*	Isolate	/

Three isolates of MTB were known to be extensively drug-resistant, as assayed using phenotypic drug susceptibility testing (pDST). Prior to this study, the species of these samples and the mutations underlying drug resistance were confirmed using Sanger sequencing. XDR^#^ of sample No.3: resistant to RIF, INH, STR, PZA, EMB, FQ, and KAN. XDR^$^ of sample No.4: resistant to RIF, INH, STR, PZA, EMB, and FQ. XDR^&^ of sample No.5: resistant to RIF, INH, STR, PZA, EMB, FQ, KAN, CAP, AMK, and aminoglycosides. XDR, extensive drug-resistant; RIF, rifampicin; INH, isoniazid; STR, streptomycin; PZA, pyrazinamide; EMB, ethambutol; FQ, fluoroquinolones; AMK, amikacin; CAP, capreomycin; KAN, kanamycin.

After nanopore sequencing, the sizes of targeted gene fragments and the typical sequencing depths of each gene were illustrated, as shown in Figures 2B,C. By analyzing the datasets of all 18 targeted genes from 10^2^ bacteria/mL to 10^7^ bacteria/mL, at a time point of 60 min the LOD of NTS reached 10^2^ bacteria/mL, as the sequencing depths of each gene were greater than 100× (Supplementary Figure S2). The drug resistance information of the MTB isolates was revealed by NTS (Figure 2D), which was found in accordance with pDST and Sanger sequencing (Table 1).

### NTS data quality

**Figure 3:**
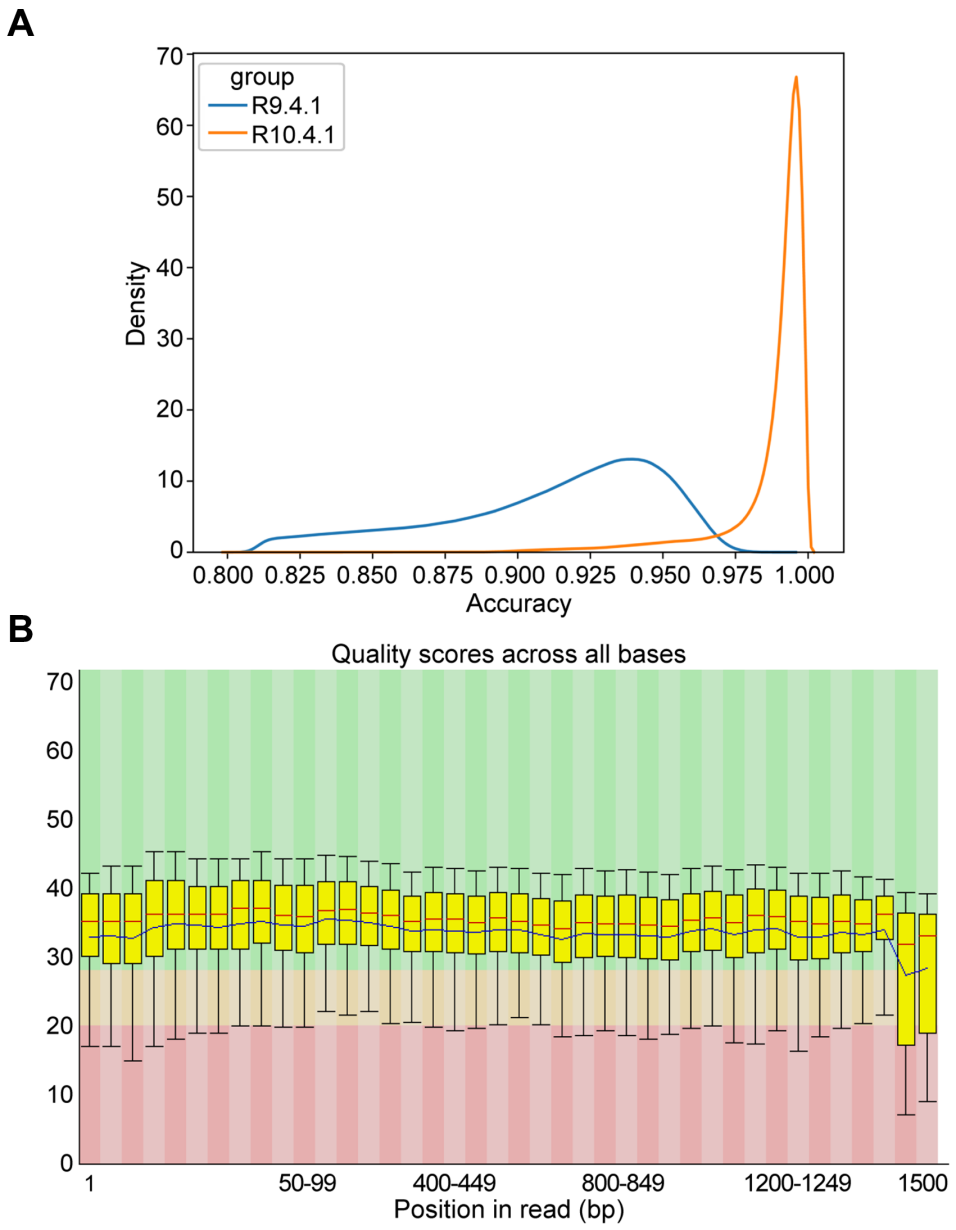
Accuracy of multiple amplicons sequenced by nanopore targeted sequencing (NTS). **(A)** The density distribution comparison of raw sequence accuracy of multiple amplicons between ONT flow cell R9.4.1 and R10.4.1. **(B)** Per base sequence quality of the single-base *Q*-value corresponding to the base position in the multiple amplicons from ONT flow cell R10.4.1. The column area of the box-whisker plot represents the range of 25–75% of *Q*-value, and the area between the upper and lower black lines represents the range of 10–90%. The blue line represents the average *Q*-value in the position, and the red line represents the median of the *Q*-value in the position. The background color divides the picture into three parts: good base quality (green), medium base quality (yellow), and poor base quality (red).

To assess sequence quality underlying NTS performance, the data from ONT flow cell R9.4.1 and R10.4.1 were reviewed, revealing that the accuracy was much improved from 94% in R9.4.1 to 99% in R10.4.1 (Figure 3A). As Q20 represents the probability that base detection error is 1%, the *Q* value of most bases from ONT flow cell R10.4.1 data was basically above 20 with low fluctuation (Figure 3B), which was in agreement with the report (Sereika et al., 2022). To ensure stable and high-quality data, ONT flow cell R10.4.1 and its ancillary kits were only used subsequently in this study, and 60 min of sequencing time was adequate.

### NTS for clinical MTB detection

**Figure 4:**
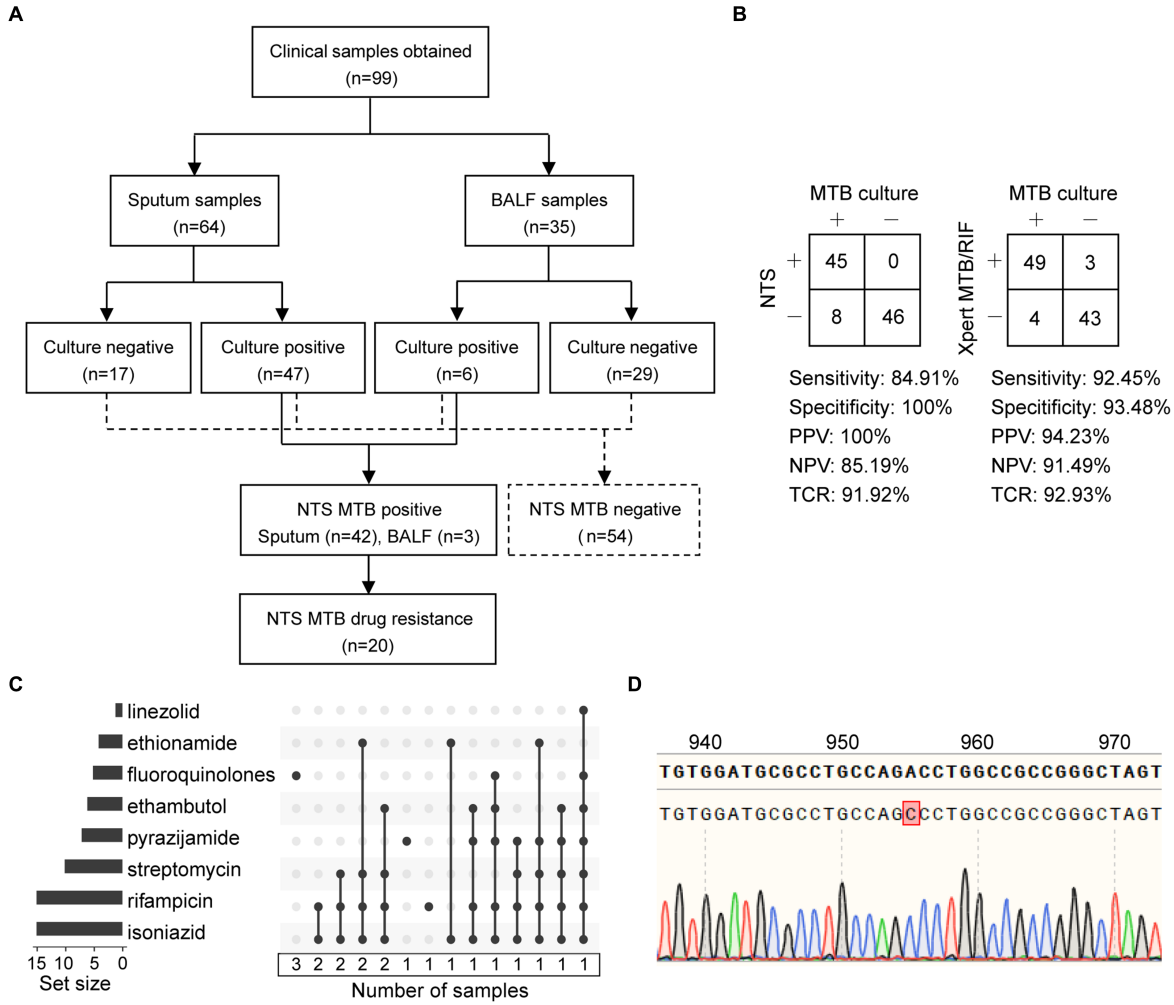
Statistics of clinical samples and their processes of inclusion in nanopore targeted sequencing (NTS) assay. **(A)** Analysis workflow of the 99 clinical samples included in the study. **(B)** Contingency tables of MTB culture with NTS and Xpert MTB/RIF sets. Abbreviations: PPV, positive predictive value; NPV, negative predictive value; TCR, the total coincidence rate. **(C)** NTS-based drug resistance data are available for clinical samples after using the TBProfiler, with a total of 20 drug-resistant samples identified. Each row is a drug, and the columns with filled cells represent the set of samples possessing drug resistance information. The box below shows the number of samples in the set. The bar plot in the left panel shows the number of samples with resistance information for each drug. **(D)** The mutation at position 955 of the *embB* gene is detected using NTS and verified using Sanger sequencing.

As shown in Figure 4A and Supplementary Table S2, a total of 99 patients were enrolled with their clinical samples subjected to Xpert MTB/RIF and pDST, followed by NTS, in this retrospective study. Multiplex PCR-based amplicons from the clinical samples could be visualized using agarose gel electrophoresis (Supplementary Figure S3).

Considering MTB culture as a reference standard, the clinical sensitivity of NTS and Xpert MTB/RIF assays for detecting MTB was 84.91% (45/53) and 92.45% (49/53), and the clinical specificity was 100% (46/46) and 93.48% (43/46), respectively (Figure 4B). Furthermore, contingency tables also showed that the positive predictive value (PPV), negative predictive value (NPV), and total coincidence rate (TCR) of NTS were 100% (45/45), 85.19% (46/54), and 91.92% (91/99), and those of Xpert MTB/RIF were 94.23% (49/52), 91.49% (43/47), and 92.93% (92/99). The TCR results indicated that these two assays were comparable for MTB detection.

### NTS for clinical drug resistance assay

In this study, six main anti-TB drugs were available for pDST, namely rifampicin, isoniazid, ethambutol, streptomycin, amikacin, and FQ. As reported, Xpert MTB/RIF could only detect the RIF resistance in an 81-bp rifampin-resistance determining region (RRDR) of rpoB (Wu et al., 2023). NTS provided the drug resistance information related to 14 anti-TB drugs, namely rifampicin, isoniazid, streptomycin, ethambutol, pyrazinamide, FQ, amikacin, capreomycin, kanamycin, ethionamide, linezolid, clofazimine, bedaquiline, and aminoglycosides (Supplementary Figure S4).

As shown in Figures 4A,C and Table 2, a total of 20 drug-resistant samples were identified from the 99 clinical samples after the TBProfiler variant calling process, and their drug resistance data revealed that these drug-resistant cases were resistant to rifampicin (*n* = 15), isoniazid (*n* = 15), streptomycin (*n* = 10), pyrazinamide (*n* = 7), ethambutol (*n* = 6), FQ (*n* = 5), ethionamide (*n* = 4), and linezolid (*n* = 1), respectively. According to categories from the review (Singh and Chibale, 2021), these cases were XDR-TB (*n* = 2), MDR-TB (*n* = 12), isoniazid-resistant (HR)-TB (*n* = 1), and single drug-resistant (SDR)-TB (*n* = 5).

**Table 2:** Drug-resistant cases of *Mycobacterium tuberculosis* identified from the enrolled patients using the nanopore targeted sequencing assay, compared with those using phenotypic drug susceptibility testing and Xpert MTB/RIF.

Cases no.	Resistance type (NTS)	Drug-resistant (NTS)	Resistance-conferring gene and variant	pDST	Xpert MTB/RIF
1	MDR	RIF	*rpoB* His445Leu	RIF, INH	RIF
		INH	*katG* Ser315Thr		
2	MDR	RIF	*rpoB* Ser441Gln	RIF, INH, STR	RIF
		INH	*katG* Ser315Thr		
		STR	*rpsL* Lys43Arg		
		EMB	*embB* Asp354Ala		
3	MDR	RIF	*rpoB* Ser450Leu	RIF, INH, STR	RIF
		INH, ETH	*inhA* Ser94Ala		
			*fabG1* C(−15)T		
		STR	*rrs* 514A > C		
		PZA	*pncA* Cys72Tyr		
4	MDR	RIF	*rpoB* Ser450Leu	RIF, INH, STR	RIF
		INH	*katG* Ser315Thr		
		STR	*rpsL* Lys88Arg		
5	XDR	RIF	*rpoB* Asp435Val	RIF, INH, STR,	/
		INH	*katG* Ser315Thr	EMB	
		STR	*rpsL* Lys43Arg		
		EMB	*embB* Asp328Tyr		
		PZA	*pncA* Val139Ala		
		FQ	*gyrA* Ser91Pro		
		LZD	*rplC* Cys154Arg		
6	MDR	RIF	*rpoB* His445Asn	RIF, INH, STR	RIF
		INH	*katG* Ser315Thr		
		STR	*rrs* 514A > C		
		PZA	*pncA* Thr114Ala		
7	XDR	RIF	*rpoB* Ser450Leu	RIF, INH, FQ	RIF
		INH	*katG* Ser315Thr		
		EMB	*embB* Gly406Asp		
		PZA	*pncA* Arg154Gly		
		FQ	*gyrA* Ser95Thr		
8	MDR	RIF	*rpoB* His445Leu	RIF, INH, STR	RIF
		INH	*katG* G(−7)A		
			*fabG1* C(−15)T		
		STR	*rpsL* Lys43Arg		
		ETH	*fabG1* C(−15)T		
9	MDR	RIF	*rpoB* His445Asp	RIF, INH, STR	RIF
		INH	*katG* Ser315Thr		
		STR	*rpsL* Lys43Arg		
10	SDR	RIF	*rpoB* His445Asp	RIF	RIF
11	HR	INH, ETH	*fabG1* -15C > T	INH	/
12	MDR	RIF	*rpoB* Ser450Leu	RIF, INH, STR	RIF
		INH	*katG* Ser315Thr		
		STR	*rpsL* Lys43Arg		
		EMB	*embB* Gly406Ser		
13	MDR	RIF	*rpoB* Ser450Leu	RIF, INH	RIF
		INH	*katG* Trp91Arg		
14	MDR	RIF	*rpoB* Ser450Leu	RIF, INH, STR	/
		INH	*katG* Ser315Thr		
		STR	*rpsL* Lys43Arg		
		EMB	*embB* Met306Val		
		PZA	*pncA* Val7Gly		
15	MDR	RIF	*rpoB* Ser450Leu	RIF, INH, STR	RIF
		INH, ETH	*fabG1* C(−15)T		
		STR	*rpsL* Lys43Arg		
16	MDR	RIF	*rpoB* Asp435Val	RIF, INH	RIF
			*rpoB* His445Asn		
		INH	*katG* Pro398Ser		
		EMB	*embB* Met306Ile		
		PZA	*pncA* Gly97Asp		
17	SDR	PZA	*pncA* Leu120Arg	/	/
18	SDR	FQ	*gyrB* Arg446Cys	/	/
19	SDR	FQ	*gyrA* Ala90Val	/	/
20	SDR	FQ	*gyrB* Ala504Thr	/	/

NTS, nanopore targeted sequencing; MTB, *Mycobacterium tuberculosis*; pDST, phenotypic drug susceptibility testing; MDR, multidrug-resistant; XDR, extensive drug-resistant; HR, isoniazid-resistant; SDR, single drug-resistant; RIF, rifampicin; INH, isoniazid; STR, streptomycin; EMB, ethambutol; PZA, pyrazinamide; FQ, fluoroquinolones; ETH, ethionamide; LZD, linezolid.

Compared to Xpert MTB/RIF, NTS was not limited to hotspot mutations, providing much more information on drug resistance (Table 2). The concordance between pDST and NTS from variant calls of rifampicin, isoniazid, streptomycin, and FQ was 100%. For ethambutol, the drug resistance rate detected by NTS was higher than that of pDST. Assaying 14 anti-TB drugs simultaneously without the restriction of drugs on hand (Supplementary Figure S4), NTS had many more advantages over pDST, beyond just efficiency. The mutations related to drug resistance from NTS were all inspected using Sanger sequencing and found to have a 100% concordance rate between the two methods, for instance, an A > C mutation at position 955 of *embB* detected by NTS and verified using Sanger sequencing (Figure 4D).

## Discussion

In this study, NTS was established for MTB drug resistance assay with a TAT of approximately 7.5 h, which was efficient and significantly shorter than that of MTB culture (Koch et al., 2018). Our NTS approach integrated the properties of single-tube multiplex PCR, long-read nanopore sequencing with the latest flow cell, bioinformatic pipeline TBProfiler, and fast DNA extraction. The analytical and clinical performances demonstrated that NTS for MTB identification and drug resistance analysis was promising for clinical use, which would promote timely susceptibility-directed antibiotic therapy (Dippenaar et al., 2022).

Although NTS yielded a read depth of each gene over 100× per sample, it was inconsistent across the 18 targeted genes, due to amplification bias during multiplex PCR. Optimizing the sequence and concentration of each primer pair in the primer mixture can improve the performance of the output data. The high G + C rate and homopolymer regions of MTB DNA might lead to errors during gene recognition (Chitale et al., 2022), which undoubtedly brought great challenges to nanopore sequencing (Gomez-Gonzalez et al., 2022; Sereika et al., 2022). By using the recently updated R10.4.1 version of ONT flow cells with a longer barrel and a dual reader head in the sequencing pores, the sequencing accuracies and qualities of NTS were improved (Sereika et al., 2022). PromethION could be replaced with other ONT devices, such as the portable MinION, and a Flongle flow cell was also an option.

The TCR of NTS for MTB detection was comparable to that of Xpert MTB/RIF, which is a well-known real-time PCR-based rapid assay, but NTS provided abundant information on MTB drug resistance. The 18 targeted genes covered most of the discovered mutations related to MTB drug resistance, which were reported in our previous studies (Li et al., 2020; Wan et al., 2020; Li et al., 2021; Liang et al., 2021; Shi et al., 2022), and other researchers’ investigations, especially using tNGS (Zhao et al., 2014; Cabibbe et al., 2020; Tafess et al., 2020; Wang et al., 2021; Dippenaar et al., 2022; Liu et al., 2022; Murphy et al., 2023). Furthermore, TBProfiler was introduced with its database containing 1,195 candidate mutations, which were obtained globally from more than 17,000 clinical isolates and assessed with whole-genome sequencing (WGS) and pDST (Phelan et al., 2019). Compared to WGS, which can sufficiently detect MTB genotypes, NTS is not limited to hotspot mutations. NTS offers greater sequencing depth with reduced costs and data burden.

In our study, codon 450 and codon 445 were the most common mutation sites in the *rpoB* gene for RIF resistance. *katG* S315T and *fabG1* C(−15)T were the most common INH resistance mutation sites. Ethambutol resistance-associated mutations occurred majorly in *embB* M306V, streptomycin resistance-associated variants could be found in *rpsL* K43R and *rpsL* K88R, and pyrazinamide resistance-associated mutations scattered throughout the *pncA* gene and its promoter. For second-line anti-TB drugs, FQ resistance-associated mutations were in either subunit of DNA gyrase (*gyrA* or *gyrB*), and *rplC* C154A was associated with linezolid resistance. NTS provided drug resistance information related to 14 anti-TB drugs, while pDST only included 6 main anti-TB drugs because other anti-TB drugs were not routinely performed. These assay results were in agreement with previous reports (Zhao et al., 2014; CRyPTIC Consortium and the 100,000 Genomes Project et al., 2018; Zaw et al., 2018; Li et al., 2020; Riviere et al., 2020; Li et al., 2021; Liang et al., 2021; Wang et al., 2021; Liu et al., 2022).

Our study had some limitations. First, due to the possibility of DNA degradation and DNA not being pure enough for multiplex PCR, the MTB detection rate of NTS was not very high. Second, the number of enrolled patients was no more than 100, and there was no case identified to be resistant to amikacin, kanamycin, capreomycin, aminoglycosides, bedaquiline, or clofazimine. Third, the drug resistance assay might be affected by bacterial lineage, which was not taken into consideration. Further investigation would be needed to expand the clinical sample size in our next study, as well as the prospective and multicenter scope to consolidate the NTS performance.

As the global prevalence of drug-resistant TB is very high (Koch et al., 2018; Huang and Zhao, 2022; Salari et al., 2023), our NTS approach has the potential to speed up the diagnosis, which would help clinical patient management and infection control to prevent a wider spread of TB and reduce the mortality rate.

## Data availability statement

The data presented in this study were deposited to the NCBI SRA repository, accession number SRP508544.

## Ethics statement

The studies involving humans were approved by the Research Ethics Board of Wenzhou Central Hospital. The studies were conducted in accordance with the local legislation and institutional requirements. Written informed consent for participation in this study was provided by the participants’ legal guardians/next of kin.

## Author contributions

CT: Writing – original draft, Visualization, Validation, Investigation, Formal analysis, Data curation. LW: Writing – review & editing, Validation, Resources, Investigation, Formal analysis, Data curation. ML: Writing – review & editing, Validation, Resources, Investigation, Formal analysis, Data curation. JD: Writing – review & editing, Validation, Resources, Formal analysis. YS: Writing – review & editing, Investigation, Formal analysis, Data curation. QW: Writing – review & editing, Visualization, Software, Data curation. FX: Writing – review & editing, Validation, Methodology, Formal analysis. LZ: Writing – review & editing, Resources, Investigation, Formal analysis, Data curation. XX: Writing – review & editing, Visualization, Software, Formal analysis. JC: Writing – review & editing, Investigation, Data curation. YZ: Writing – review & editing, Investigation, Data curation. YY: Writing – review & editing, Investigation, Data curation. XZ: Writing – review & editing, Supervision, Resources, Conceptualization. GX: Writing – original draft, Visualization, Supervision, Resources, Project administration, Methodology, Funding acquisition, Formal analysis, Conceptualization.

## Footnotes

^1^ https://www.mfeprimer.com/

^2^ https://github.com/jodyphelan/TBProfiler
