# Supplementary material for: High-throughput nanopore targeted sequencing for efficient drug resistance assay of Mycobacterium tuberculosis
Source: Front Microbiol. 2024 May 22;15:1331656. doi: 10.3389/fmicb.2024.1331656 (PMC11152171; doi:10.3389/fmicb.2024.1331656)
Supplement: Supplementary file 1 [file Data_Sheet_1.PDF]

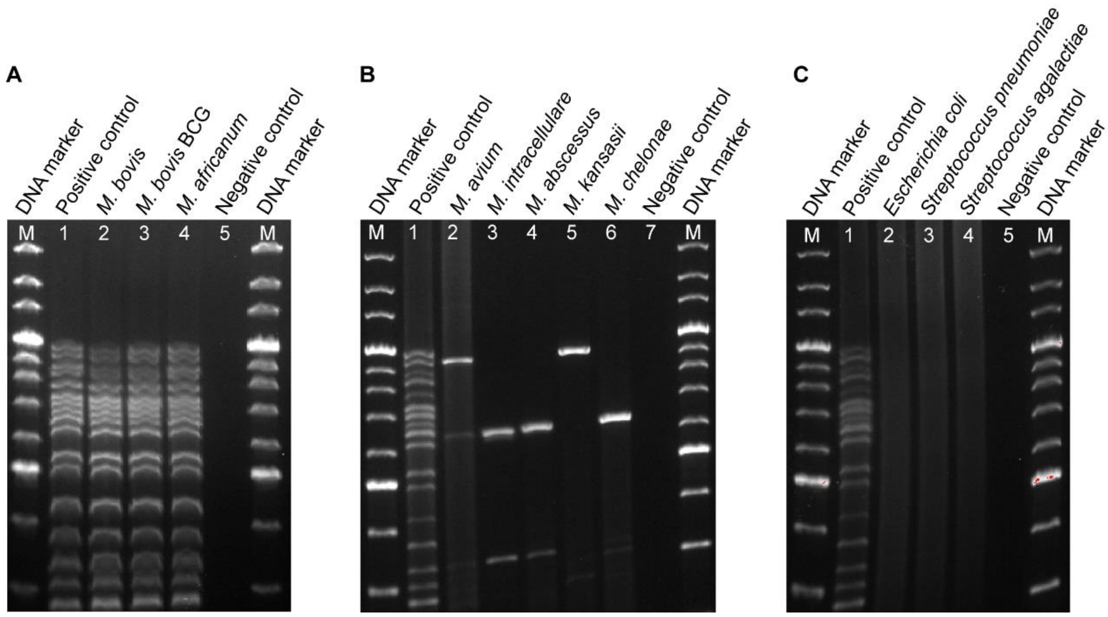

Figure S1. Electrophoresis patterns of multiplex PCR-based amplicons. The ladder sizes of DNA markers (M) were from 600 bp to 4000 bp. The positive control was the reference strain *Mycobacterium tuberculosis* (MTB) H37Rv, and the negative control was nuclease-free water. The bands of electrophoretic lane 1 (positive control) from bottom to top were *rpsL* (560 bp), *mmpR5* (600 bp), *rplC* (652 bp), *atpE* (744 bp), *tlyA* (827 bp), *pncA* (971 bp), *ubiA* (1041 bp), *gyrB* (1210 bp), *rrl* (1275 bp), *eis* (1322 bp), *fabG1* & *inhA* (1387 bp), *rpsA* (1444 bp), *gyrA* (1544 bp), *katG* (1653 bp), *rrs* (1741 bp), *rpoB* (1845 bp), and *embB* (1925 bp), respectively. **(A)** Electrophoresis patterns of amplicons from four species of *Mycobacterium tuberculosis* complex, including MTB, *Mycobacterium bovis*, *Mycobacterium bovis* BCG and *Mycobacterium africanum*, from lane 1 to lane 4, respectively. **(B)** Electrophoresis patterns of amplicons from five strains of non-tuberculous mycobacteria species, including *Mycobacterium avium*, *Mycobacterium intracellulare*, *Mycobacterium abscessus*, *Mycobacterium kansasii*, and *Mycobacterium chelonae*, from lane 2 to lane 6, respectively. **(C)** Electrophoresis patterns of amplicons from three other bacteria, including *Escherichia coli*, *Streptococcus pneumoniae* and *Streptococcus agalactiae*, from lane 2 to lane 4, respectively.

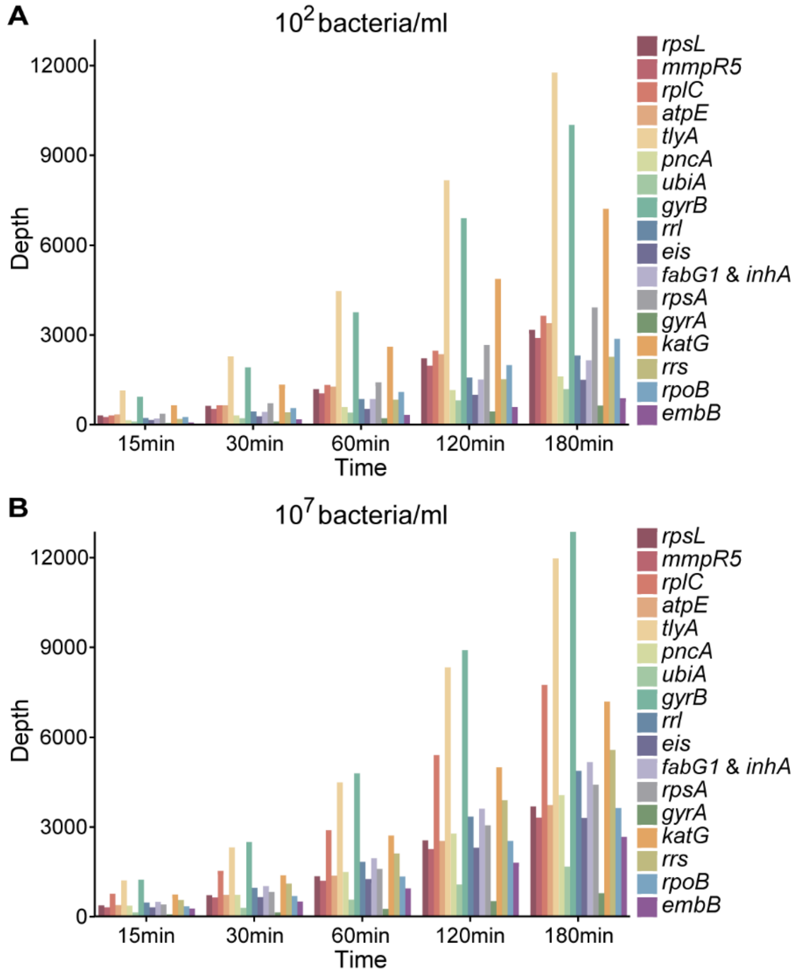

Figure S2. Performance of nanopore targeted sequencing for drug resistance assay of *Mycobacterium tuberculosis* (MTB) with sequencing depths at different concentrations. Reads were mapped to 18 targeted regions of the MTB genome and converted into sequencing depths with 10^2 bacteria/mL **(A)** and 10^7 bacteria/mL **(B)** concentrations at 5 different time points.

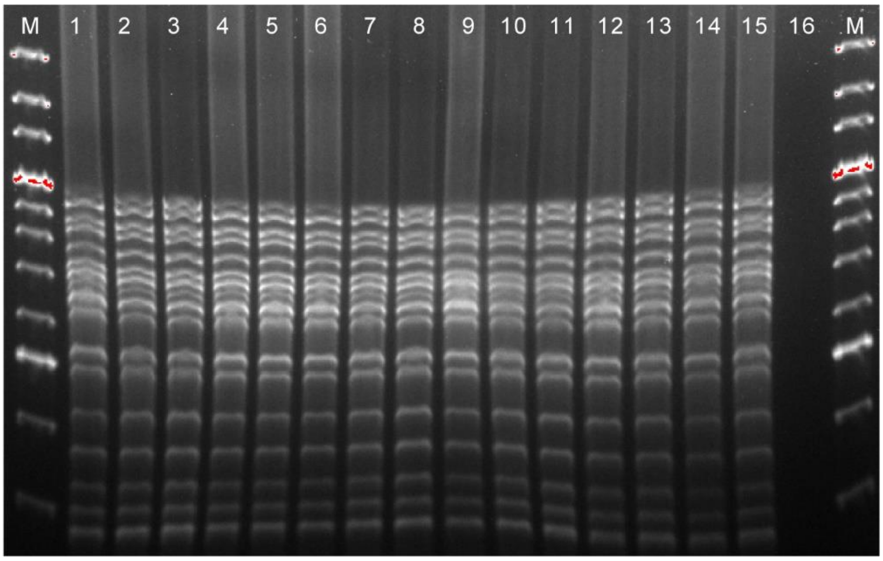

Figure S3. Electrophoresis patterns of multiplex PCR-based amplicons from clinical samples. Amplicons of clinical samples with or without *Mycobacterium tuberculosis* were electrophoresed from lane 1 to lane 16, along with DNA markers (M). The ladder sizes of DNA markers were from 600 bp to 4000 bp. The bands of electrophoretic lane 1-15 from bottom to top were *rpsL* (560 bp), *mmpR5* (600 bp), *rplC* (652 bp), *atpE* (744 bp), *tlyA* (827 bp), *pncA* (971 bp), *ubiA* (1041 bp), *gyrB* (1210 bp), *rrl* (1275 bp), *eis* (1322 bp), *fabG1* & *inhA* (1387 bp), *rpsA* (1444 bp), *gyrA* (1544 bp), *katG* (1653 bp), *rrs* (1741 bp), *rpoB* (1845 bp), and *embB* (1925 bp), respectively.

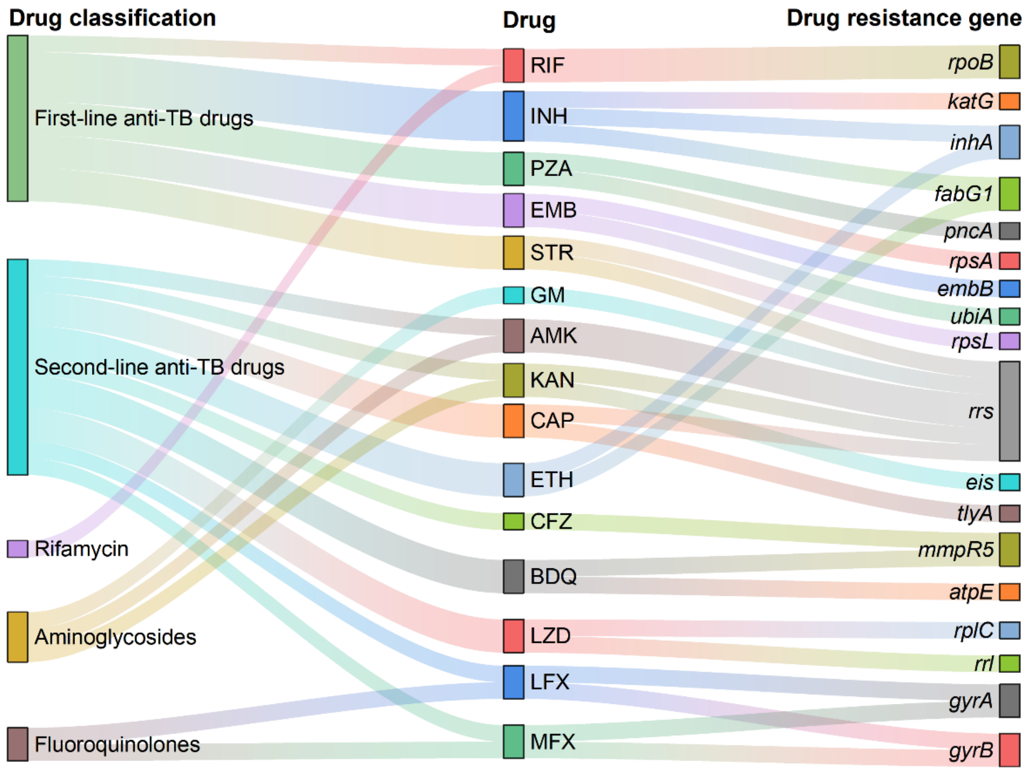

Figure S4. The drug resistance information of nanopore targeted sequencing related to 14 anti-tuberculosis (TB) drugs. Sankey diagram described the corresponding relationship between anti-TB drugs and drug resistance associated genes in this study. Abbreviations: RIF, rifampicin; INH, isoniazid; PZA, pyrazinamide; EMB, ethambutol; STR, streptomycin; GM, gentamycin; AMK, amikacin; KAN, kanamycin; CAP, capromycin; ETH, ethionamide; CFZ, clofazimine; BDQ, bedaquiline; LZD, linezolid; LFX, levofloxacin; MFX, moxifloxacin. LFX and MFX belong to fluoroquinolones.

Table S1. Primer sequences used for multiplex PCR.

No.	Gene	Primer sequence (5'-3')	Start-end location	Amplicon size	Working concentration
1	*rpsL*	AGGCAAGCTATGCGACACAC<br>AGAACCTTGTTCACCAACTGGG	781466-782025	560 bp	0.045 μM
2	*mmpR5*	TCTGGTGACGCATACCGAACG<br>ACAGGCCAATCGCCGTCTT	778941-779540	600 bp	0.03 μM
3	*rplC*	TGGCACGAAAGGGCATTCT<br>CACTTCTCACCTCGTTTGATCG	800810-801461	652 bp	0.035 μM
4	*atpE*	AGAAGTCGCCAAGCCAATCT<br>ACCATAGCGTCACGTTCCC	1460768-1461511	744 bp	0.035 μM
5	*tlyA*	GGAGAAGGGTTGAGTGCGG<br>CTACGGGCCCTCGCTAATC	1917920-1918746	827 bp	0.045 μM
6	*pncA*	TCTGTCACCGGACGGATTTG<br>TGCCGGAGACGATATCCAGA	2288450-2289420	971 bp	0.035 μM
7	*ubiA*	GGAGGACGTTGAGCTTGAGG<br>GGTCCACTACCCCAGTGATG	4268905-4269945	1041 bp	0.04 μM
8	*gyrB*	TTCCTCAACAAGGGGCTGAC<br>TGATCTTCTTCCCGGCCTTC	5834-7043	1210 bp	0.04 μM
9	*rrl*	ACGAGTGATGTCGTGCTACC<br>CCTTGTCGCTACTCATGCCT	1473791-1475065	1275 bp	0.08 μM
10	*eis*	GCCAGACACTGTCGTCGTAA<br>CTGACCACGCCGAAAAGC	2714075-2715396	1322 bp	0.05 μM
11	*fabG1* & *inhA*	CACGTCTTTATGTAGCGCGACA<br>ATCGAAGCATACGAATACGCCG	1673204-1674590	1387 bp	0.05 μM
12	*rpsA*	ATCATCAAGGTGGCCATGATCC<br>CGATGTCGATGACCTTGACCAT	1833152-1834595	1444 bp	0.05 μM
13	*gyrA*	CAGCGCAGCTACATCGACTA<br>CGTTTCTGGCTGCGATACAG	7374-8917	1544 bp	0.1 μM
14	*katG*	AGGACACTTTGATGTTCCCCG<br>ACAGCCCGATAACACCAACT	2154494-2156146	1653 bp	0.05 μM
15	*rrs*	TTGGCCATGCTCTTGATGCC<br>GGTGGCCAACTTTGTTGTCAT	1471744-1473484	1741 bp	0.2 μM
16	*rpoB*	GTTCGAGTGGCTGATCGGTT<br>AACTTGCGCATCCGGTAGG	759968-761812	1845 bp	0.08 μM
17	*embB*	TTGGCTTTGTGTTGTCGGTG<br>CGCAAACAGGGCGAAAAAGA	4246620-4248544	1925 bp	0.2 μM

Table S2. Characteristics of all enrolled patients with their clinical samples for the drug resistance assay of *Mycobacterium tuberculosis* using nanopore targeted sequencing.

Characteristics	Value
Gender, n (%)	
Female	21 (21%)
Male	78 (79%)
Age	
Range	17-94
Median (IQR)	54 (42-67)
Treatment, n (%)	
Initial treatment	83 (84%)
Re-treatment	16 (16%)
Clinical diagnosis, n (%)	
Pulmonary tuberculosis	51 (52%)
Extrapulmonary tuberculosis	2 (2%)
Lung infection	46 (46%)
Sample type, n (%)	
BALF	35 (35%)
Sputum	64 (65%)
Detection method, n (%)	
pDST	99 (100%)
Xpert MTB/RIF	99 (100%)

Abbreviations: IQR, interquartile range; BALF, bronchoalveolar lavage fluid; pDST, phenotypic drug susceptibility testing; MTB, Mycobacterium tuberculosis; RIF, rifampicin.
